# Hybrid ultrasound and photoacoustic contrast agent designs combining metal phthalocyanines and PBCA microbubbles†

**DOI:** 10.1039/d3tb02950f

**Published:** 2024-02-09

**Authors:** Roman A. Barmin, MirJavad Moosavifar, Rui Zhang, Stephan Rütten, Sven Thoröe-Boveleth, Elena Rama, Tarun Ojha, Fabian Kiessling, Twan Lammers, Roger M. Pallares

**Affiliations:** a Institute for Experimental Molecular Imaging, RWTH Aachen University Hospital Aachen 52074 Germany rmoltopallar@ukaachen.de tlammers@ukaachen.de; b Electron Microscope Facility, RWTH Aachen University Hospital Aachen 52074 Germany; c Institute for Occupational, Social and Environmental Medicine, RWTH Aachen University Hospital Aachen 52074 Germany

## Abstract

Photoacoustic (PA) imaging is an emerging diagnostic technology that combines the penetration depth of ultrasound (US) imaging and the contrast resolution of optical imaging. Although PA imaging can visualize several endogenous chromophores to obtain clinically-relevant information, multiple applications require the administration of external contrast agents. Metal phthalocyanines have strong PA properties and chemical stability, but their extreme hydrophobicity requires their encapsulation in delivery systems for biomedical applications. Hence, we developed hybrid US/PA contrast agents by encapsulating metal phthalocyanines in poly(butyl cyanoacrylate) microbubbles (PBCA MB), which display acoustic response and ability to efficiently load hydrophobic drugs. Six different metal chromophores were loaded in PBCA MB, showing greater encapsulation efficiency with higher chromophore hydrophobicity. Notably, while the US response of the MB was unaffected by the loading of the chromophores, the PA characteristics varied greatly. Among the different formulations, MB loaded with zinc and cobalt naphthalocyanines showed the strongest PA contrast, as a result of high encapsulation efficiencies and tunable optical properties. The strong US and PA contrast signals of the formulations were preserved in biological environment, as demonstrated by *in vitro* imaging in serum and whole blood, and *ex vivo* imaging in deceased mice. Taken together, these findings highlight the advantages of combining highly hydrophobic PA contrast agents and polymeric MB for the development of contrast agents for hybrid US/PA imaging, where different types of information (structural, functional, or potentially molecular) can be acquired by combining both imaging modalities.

## Abstract 2

The combination of polymeric PBCA microbubbles and highly hydrophobic metal phthalocyanines provides bimodal ultrasound/photoacoustic imaging capabilities based on the high encapsulation efficiencies and tunable optical properties of the latter.

## Graphical abstract

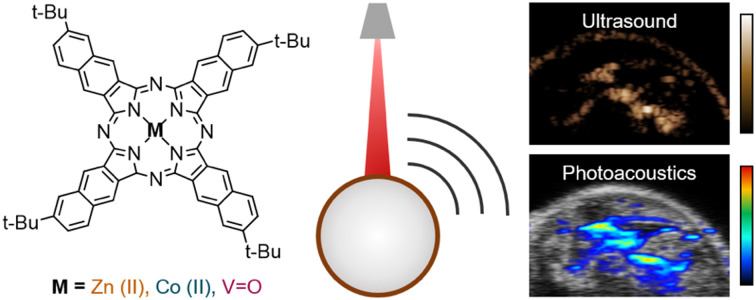

## 1. Introduction

Photoacoustic (PA) imaging is a non-ionizing imaging modality based on the conversion of light energy into sound by an absorber *via* the photothermal effect.^1,2^ PA imaging combines the high contrast of optical imaging with the high spatial resolution of ultrasound (US), as the US transducer detects generated sound pressure waves and reconstructs an image.^3^ While conventional US imaging (without administration of contrast agents) provides anatomical information, PA imaging is capable of providing molecular information by imaging endogenous chromophores, such as hemoglobin, melanin, lipids, and collagen. For instance, the unique and distinct PA features of oxy- and deoxyhemoglobin are exploit in the clinic for oxygen saturation measurements and vascular angiography.^4,5^ Nevertheless, since only a few biomolecules can provide characteristic absorption features adequate for PA, the list of suitable endogenous chromophores for disease diagnosis is very limited. In addition, a strong background signal from dozens of non-specific endogenous chromophores present in tissues can hinder the use of PA in molecular imaging.^6,7^

To overcome this problem, exogenous contrast agents with higher absorption coefficients than endogenous counterparts have been developed. Some examples include organic dyes, such as indocyanine green and methylene blue, and metallic or semiconducting nanostructures.^6–9^ Nevertheless, organic dyes are limited by their rapid photobleaching, irreversible aggregation in aqueous media, and non-specific protein interactions in biological fluids, which can drastically disrupt their absorption spectra and reduce their PA contrast capabilities.^10–12^ Metallic nanoparticles, on the other hand, do not photobleach, and thus their PA signal is preserved over time. However, their tendency to accumulate in fenestrated organs, such as liver and spleen, for prolonged periods of time limits their clinical use^13^ and has promoted new efforts to develop formulations with improved accumulation profiles.^14,15^ Metal phthalocyanines (MePc), naphthalocyanines (MeNc) and their derivatives exhibit intense absorbance in the red and near-infrared regions of the spectrum, where light displays deeper tissue penetration, as well as high chemical stability, resulting in their use as PA contrast agents and sensitizers for photo-/sono-dynamic therapy.^16–19^ The two central hydrogen atoms of their tetrapyrrolic macrocycle can be substituted by a wide range of metal elements (including zinc, cobalt, and vanadyl), affecting chromophore absorbance and solvent solubility.^20,21^ However, their extreme hydrophobicity and poor solubility in aqueous solutions require their encapsulation into delivery systems.^16,21–23^

Micron-sized microbubbles (MB) made of poly(butyl cyanoacrylate) (PBCA) polymer can serve as effective carriers for encapsulating hydrophobic molecules.^24,25^ Moreover, the MB air-filled core displays acoustic responsiveness, leading to its primary use in US contrast imaging.^26^ The PBCA MB platform is biocompatible at diagnostic doses, and can be bioconjugated with targeting agents, expanding its use to molecular imaging and targeted drug delivery, among others.^24,27,28^ In a previous study, we demonstrated the good loading of hydrophobic molecules with different chemical structures, namely nile red (log *P* value of 3.5), coumarin 6 (log *P* 4.9), and pyrene (log *P* 6.0) in PBCA MB, and their US-induced release.^29^ In addition, the signal of PA agents (*i.e.*, porphyrins and gold nanoparticles) has been recently enhanced by loading them in the shells of MB, and exploiting the radial oscillation capabilities of the latter.^30–33^ Therefore, we hypothesize that the high hydrophobicity of MePc and MeNc derivatives (with log *P* values up to 10.2) can promote their efficient encapsulation into PBCA MB shells, while their high molar extinction coefficients and tunable absorbance bands combined with the acoustic amplification provided by the MB templates can facilitate strong PA signal generation.

In this study, we developed new hybrid contrast agents for PA and US imaging by encapsulating MePc and MeNc with different chelated metals into PBCA MB. We have shown that the higher hydrophobicity of MeNc promotes higher chromophore loading into MB polymer shells compared to the moderately hydrophobic MePc. After chromophore loading, the encapsulated dyes display PA signals that are proportional to their optical properties, while the resulting PBCA MB formulations preserve their US-imaging contrast capabilities. Zinc and cobalt chelated MeNc, which show the highest levels of encapsulation into MB shells (on the order of 10^7^ molecules/MB), provide MB with strong PA signals with the highest intensity values around 770 nm. Moreover, *ex vivo* studies of selected dye-loaded specimens demonstrate high PA and US contrast signals in deceased mice. Taken together, these results prove the benefits of combining highly hydrophobic PA contrast agents and polymeric MB for the development of contrast agents for hybrid imaging, which may be beneficial to extract different information, such as anatomical, functional and molecular, and for image-guided therapy.

## 2. Materials and methods

### 2.1. Materials

Butyl cyanoacrylate (BCA) was purchased from Special Polymer Ltd (Sofia, Bulgaria). Triton X-100, zinc(ii) phthalocyanine (ZnPc), zinc(ii) 2,11,20,29-tetra-*tert*-butyl-2,3-naphthalocyanine (ZnTTBNc), cobalt(ii) phthalocyanine (CoPc), cobalt(ii) 2,3-naphthalocyanine (CoNc), vanadyl(iv) phthalocyanine (VPc), vanadyl(iv) tetra-*tert*-butyl-2,3-naphthalocyanine (VTTBNc), gelatin, and dimethyl sulfoxide (DMSO) were purchased from Sigma-Aldrich (Munich, Germany). Phosphate-buffered saline (PBS), and fetal bovine serum (FBS) were purchased from Thermo Fisher Scientific (Dreieich, Germany). Deionized (DI) water was produced by a PURELAB flex 2 device from ELGA LabWater (Celle, Germany) and used for all experiments. All reagents were of appropriate analytical grade.

### 2.2. Dye absorbance and fluorescence measurements

Absorbance and fluorescence spectroscopy were performed with a TECAN Infinite M200 Pro (TECAN group Ltd, Männedorf, Switzerland). All dyes were dissolved in DMSO or DI water (in the presence of 5% DMSO) to a concentration of 50 μg mL^−1^, and a 150 μL aliquot of each dye solution was collected in triplicate for measurements. Absorbance intensity was recorded in the 500–900 nm wavelength range. Fluorescence intensity was recorded under the excitation wavelength of 640 nm for the wavelength range of 670–850 nm. In addition, fluorescence intensity was recorded under excitation at the maximum absorption wavelengths of the chromophores listed in Table S1 (ESI†). Because the Stokes shifts of MePc and MeNc are relatively small (of 6–10 nm),^34^ detection of fluorescence within 20 nm of the maximum absorption wavelengths was challenging with our spectrometer. Therefore, a wavelength range of 20 nm from the excitation wavelength to 850 nm was considered for the measurements.

### 2.3. PBCA MB synthesis, dye loading, and destruction

PBCA MB were synthesized *via* anionic polymerization of BCA monomers in the presence of hydroxyl ions as previously described.^25,35,36^ Briefly, an aqueous solution of 1% (v/v) Triton X-100 was prepared, and the pH of the solution was adjusted to 2.5. Next, 1% (v/v) of BCA were added dropwise to the solution at room temperature, and the resulting mixture was stirred for 1 h at 10 000 rpm using an Ultra-Turrax T50 (IKA-Werke, Staufen, Germany). The produced suspension of air-filled PBCA MB was purified by three centrifugation steps at 500 rpm for 20 min and stored in a 0.02% (v/v) Triton X-100 aqueous solution at pH 7 (labeled in the manuscript as storage solution).

MePc and MeNc were encapsulated into the shell of the PBCA MB following a previously described protocol.^25,29^ 5 mg of each dye were used to ensure the maximum loading into the MB shell. The 5 mg were dissolved in 500 μL of DMSO at 50 °C. After dye dissolution, each solution was cooled down at room temperature, and mixed with 10 mL of solution containing 10^10^ MB. The mixtures were stirred at 50 rpm for 24 h. Once stirring was finished, the samples were left to rest overnight to allow the MB to float and form a MB cake. The aqueous solutions under each MB cake were replaced several times to remove the unencapsulated dye until no free drug was present in the solution and no precipitates were found on the vial bottom. As a result, dye-encapsulated MB were resuspended in 5 mL of storage solution.

To evaluate US-induced dye release, 0.1 mL of each dye-encapsulated MB suspension was mixed with 9.9 mL of storage solution, and sonicated for 10 min at 60 W in a US cleaner (Emmi H60, EMAG AG, Mörfelden-Walldorf, Germany) to ensure that all MB present in the sample were destroyed. Samples were then centrifuged at 4700 rpm for 10 min to allow MB shell fragments to precipitate. After precipitate removal, the aqueous solutions containing the released dyes were kept in the dark for characterization.

### 2.4. MB diameter distribution, concentration and absorption profile evaluation

For each MB sample, diameter distribution and concentration were quantified with a Coulter Counter Multisizer 3 (Beckmann Coulter, Krefeld, Germany). For this, 2 μL of MB solution were mixed with 20 ml of ISOTON® II (Beckman Coulter) and measured in a volumetric mode at room temperature. Wide-area optical microscopy (OM) was carried out with a Zeiss Axiovert 40 C with an LD A-Plan 40×/0.5 Ph2 objective (Carl Zeiss Microscopy GmbH, Jena, Germany) at a MB concentration of 1 × 10^8^ MB mL^−1^. Absorbance intensity of the MB samples was recorded in the 500–900 nm wavelength range using a TECAN Infinite M200 Pro (TECAN group Ltd, Männedorf, Switzerland), where a 150 μL aliquot of 1 × 10^9^ MB mL^−1^ solutions was collected in a triplicate.

### 2.5. MB morphology and shell thickness evaluation

Scanning electron cryo-microscopy (cryoSEM) was implemented to evaluate the morphology and shell thickness of plain and chromophore-encapsulated MB. CryoSEM images were obtained using an FE-SEM 4800 (Hitachi, Krefeld, Germany) having an Alto 2500 Cryo-Gatan unit (Gatan GmbH, Munich, Germany) at 1 kV and 2 μA. At least 50 separate MB were evaluated to quantify the shell thickness of each sample with ImageJ software (National Institutes of Health, USA).

### 2.6. Chromophore loading and release quantification

Inductively coupled plasma mass spectrometry measurements were performed to determine the concentrations of metal ions chelated in chromophores for intact and destroyed MB samples using the Agilent 8900 ICP-QQQ instrument (Agilent, Waldbronn, Germany). The nebulizer used was a MicroMist quartz glass Meinhard nebulizer. RF power was 1550 W, plasma gas flow was 15 L min^−1^, and nebulizer gas flow was 1.15 L min^−1^. The injector used was a 2 mm quartz glass injector.

The intact MB samples were vigorously agitated to achieve uniform distribution of MB floated in the solution. Approximately 200 mg of each sample was then removed and accurately weighed into a microwave pressure digestion vessel, followed by the addition of internal standard (Rhodium, 1 mg L^−1^, Merck, Darmstadt, Germany), 2.5 mL nitric acid (Suprapur, 65%, Merck, Darmstadt, Germany), 2.5 mL hydrochloric acid (Suprapur, 32%, Merck, Darmstadt, Germany), 2.5 mL hydrogen peroxide (Suprapur, 30%, Merck, Darmstadt, Germany) and 2.5 mL DI water. The samples were then digested in the Ethos.lab microwave pressure digestion system (MLS GmbH, Leutkirch, Germany). In the program used, the samples were first heated to 210 °C within 45 min. This temperature was maintained for 15 min and then cooled to room temperature.

For aqueous solutions obtained after US-induced dye release, 1 mL was taken and mixed with 20 μL of nitric acid (Suprapur, 65%; Merck, Darmstadt, Germany) and internal standard (rhodium, 1 mg L^−1^; Merck, Darmstadt, Germany). The volume of the mixture was adjusted to 10 mL with DI water.

For each chromophore, the number of dye molecules encapsulated per single MB was calculated according to 
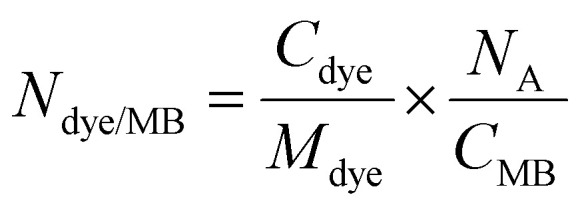
, where *C*_dye_ is the concentration of the encapsulated chromophore (mg mL^−1^), *M*_dye_ is the molecular weight of the corresponding dye (577 910 mg mol^−1^ for ZnPc, 1 002 570 mg mol^−1^ for ZnTTBNc, 571,460 mg mol^−1^ for CoPc, 771 690 mg mol^−1^ for CoNc, 579,460 mg mol^−1^ for VPc, and 1 004 120 mg mol^−1^ for VTTBNc, respectively), *N*_A_ is the Avogadro's number, and *C*_MB_ is the concentration of chromophore-encapsulated MB (number of MB mL^−1^).

US-induced chromophore release (%) was calculated as the ratio between the chromophore concentrations measured for the solutions containing the released dyes after MB destruction and the chromophore concentrations measured for chromophore-encapsulated MB samples.

### 2.7. *In vitro* US and PA imaging of MB phantoms

US imaging of plain and chromophore-encapsulated MB was performed using custom-made gelatin phantoms. Briefly, 5 × 10^5^ MB were gently dispersed in 4.5 mL of 2% (w/v) gelatin, which was embedded in 10% (w/v) gelatin bulk and kept overnight at 4 °C to solidify. A preclinical Vevo 2100 US device (VisualSonics, Amsterdam, The Netherlands) was employed to image the MB in gelatin phantoms using the non-linear contrast (NLC) mode at a center frequency of 18 MHz, initially with 4% power (corresponds to mechanical index value of 0.03), followed by a higher 10% power (mechanical index value of 0.07) applied for 5 s to destroy the MB trapped in gelatin, and then imaged again with 4% power. A region-of-interest of approximately 25 mm^2^ was drawn in the middle of all images, and the resulting NLC intensity was analyzed using the Vevo LAB software. The percentage of MB destroyed by US was quantified as the ratio between the difference in mean MB NLC signal intensity before and after the application of higher acoustic power and the initial mean MB NLC signal intensity recorded at 4% acoustic power.

PA imaging was performed using the Vevo® LAZR system (VisualSonics, Amsterdam, The Netherlands) that is built around the Vevo 2100 setup and equipped with the optical fiber mounted on either side of the MX 250 transducer using the fiber-holder included with the system, and the flashlamp pumped Q-switched Nd:YAG laser. To mimic *in vivo* vessel conditions, polyethylene tubes with the inner and the outer diameters of 0.58 and 0.96 mm, respectively (Reichelt Chemietechnik, Heidelberg, Germany), were fixed in the sample holder and filled with 20 μL of chromophore-encapsulated MB samples diluted to the concentrations of 1 × 10^9^ MB mL^−1^. Before the imaging session, the sample holder was filled with DI water and set at a focus depth of 10 mm. The PA spectra were acquired from 680 nm to 920 nm with a PA gain of 45 dB in triplicate. As control groups, chromophores were dissolved in DMSO or DI water at concentrations corresponding to the loading amounts in 1 × 10^9^ MB and imaged similarly to the procedure described above. All PA imaging experiments were performed with an imaging depth of 10 mm.

The PA signal stability of the selected MB samples was recorded at 680 nm under continuous laser pulse exposure every 200 ms for 3 min.

To investigate the behavior of chromophore-loaded MB in biological samples, *in vitro* PA imaging was performed in the presence of 10% FBS, 50% FBS, and 50% blood. Briefly, the MB samples were transferred to PBS and mixed with the desired amount of biological sample to achieve a MB concentration of 1 × 10^9^ MB mL^−1^, followed by PA imaging as described above. The stability of the PA signal was measured at 0 min, 10 min, 30 min, 2 h, 6 h and 24 h for solutions containing FBS and at 0 min, 5 min and 10 min for solutions containing blood. For the blood PA imaging, a mixture of plain MB with a concentration of 1 × 10^9^ MB mL^−1^ and 50% blood were taken as the control group. Measurements were performed in triplicate.

### 2.8. *Ex vivo* US and PA imaging

Combined *ex vivo* US and PA imaging on mice legs was performed using the Vevo 2100 US device equipped with Vevo® LAZR system (VisualSonics, Amsterdam, The Netherlands) and the MX 250 transducer with a center frequency of 18 MHz. The BALB/c mouse cadavers were reused from a previous experiment, and, therefore, no live animals were needed or euthanized for the current study.

Prior to imaging, the mouse legs were shaved. Before intramuscular MB administration, control images were acquired using 4% acoustic power and a US gain of 8 dB for US NLC mode and a PA gain of 39 dB, wavelength range from 680 to 920 nm, for PA spectral imaging. The settings were adjusted to minimize non-specific signals that could come from the skin surface. After recording the control images, 1 × 10^9^ MB of selected samples were injected in each leg, followed by a 50 μL saline flush, and US and PA images were recorded at nearly similar transducer positions as for the control images and similar setup settings as for controls. For each sample, 4 mouse legs were imaged before and after MB injection.

In addition, a four times lower dose of ZnTTBNc encapsulated MB (2.5 × 10^8^ MB) was injected to evaluate the US and PA imaging capabilities at lower concentrations. US NLC and PA images were recorded following the same protocol as described before.

### 2.9. Statistical analysis

Three different batches of dye-encapsulated MB were prepared for each dye, and each formulation was measured three times. All values are presented as mean ± standard deviation. Statistical analyses were performed using GraphPad Prism 8. Fig. 2, 3, 5 and 6 were analyzed using one-way ANOVA with *post hoc* Tukey HSD test. A *p* value of less than 0.05 was considered to be statistically significant, and (*) indicates groups that are significantly different with *p* < 0.05.

## 3. Results and discussion

### 3.1. MePc and MeNc selection and encapsulation into PBCA MB

**Fig. 1:**
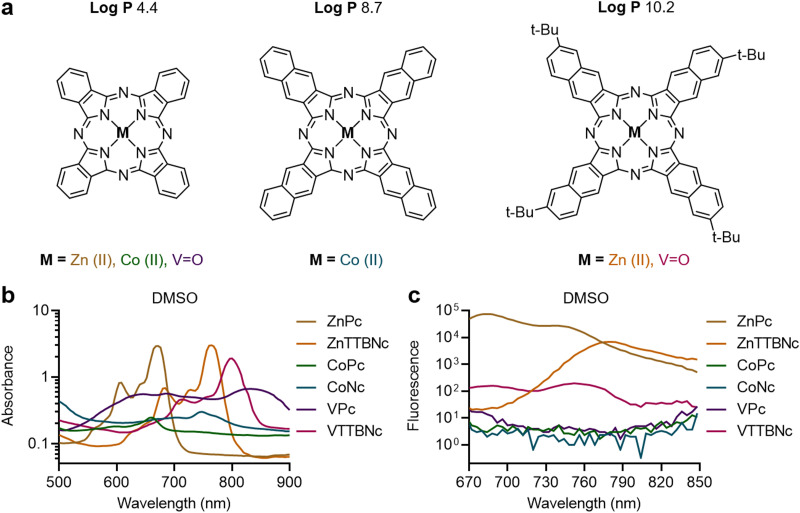
Chemical structures and optical properties of metal phthalocyanines and naphthalocyanines. (a) Commercially available phthalocyanines chelated with zinc(ii), cobalt(ii) and vanadyl(iv) (referred to as ZnPc, CoPc and VPc, respectively), cobalt(ii) naphthalocyanine (CoNc), and tetra-*tert*-butyl-2,3-naphthalocyanines chelated with zinc(ii) and vanadyl(iv) (ZnTTBNc and VTTBNc, respectively) were selected based on their high hydrophobicity (log *P*) values, which primarily depend on the chromophore structure. Variations in the chelated metals (zinc, cobalt, vanadyl) tune the optical properties of the complex. (b) Absorption spectra, and (c) fluorescence emission spectra of the different chromophores in DMSO. Fluorescence spectra were recorded upon excitation at 640 nm. Literature values of maximum absorption wavelengths, molar absorption coefficients, and fluorescence quantum yields of the chromophores are listed in Table S1 (ESI†). Absorption spectra of the chromophores in DI water, and fluorescence emission spectra of the chromophores in DMSO upon excitation at their maximum absorption wavelengths are shown in Fig. S1 (ESI†).

Six commercially available MePc and MeNc were selected to assess the effect of chromophore hydrophobicity and chelated metal on chromophore encapsulation efficiency in PBCA MB shells, and PA performance of the resulting formulation. The chemical structures of the selected dyes are shown in Fig. 1(a), and include phthalocyanines (log *P* value of 4.4) chelated with zinc(ii), cobalt (ii) and vanadyl(iv) ions (referred to as ZnPc, CoPc and VPc, respectively), naphthalocyanine (log *P* value of 8.7) chelated with cobalt(ii) (referred to as CoNc), and tetra-*tert*-butyl-2,3-naphthalocyanines (log *P* values of 10.2) chelated with zinc(ii) and vanadyl(iv) (referred to as ZnTTBNc and VTTBNc, respectively). Fig. 1(b) displays the absorbance spectra of the different dyes dissolved in DMSO and recorded at a concentration of 50 μg mL^−1^. For instance, MePc dyes chelated with zinc(ii) and cobalt(ii) showed absorbance bands in the red region of the spectrum and centered at 672 and 660 nm, respectively, whereas MeNc-based compounds displayed absorbance bands shifted to the near-infrared region (namely 764 nm for ZnTTBNc, 748 nm for CoNc, and 800 nm for VTTBNc, respectively). The position of the absorption bands were consistent with previous reports, summarized in Table S1 (ESI†), and depended on the aromaticity degree of the chromophores.^20,34,37–41^ VPc had a broader absorption band, which we hypothesize was caused by its high propensity to form dimeric structures.^42,43^ Regarding absorbance intensities of described bands, zinc-chelated chromophores showed the highest values (up to 3 a.u.), vanadyl-chelated structures showed intermediate values, while the lowest intensities were observed for cobalt-chelated ones (as low as 0.3 a.u.). When the selected chromophores were dissolved in DI water at a concentration of 50 μg mL^−1^, broadening of the absorption bands as well as drastic decrease in absorbance intensities were observed, as shown in Fig. S1a (ESI†), consistent with the tendency of MePc and MeNc to aggregate rapidly in aqueous-rich environments.^44,45^ The fluorescence spectra of the different chromophores are shown in Fig. 1(c), where all dyes were excited at 640 nm. ZnPc, ZnTTBNc, and VTTBNc showed significant fluorescence emissions, which were proportional to their molar absorption coefficients, and fluorescence quantum yields presented in Table S1 (ESI†). In contrast, CoPc, CoNc, and VPc showed no fluorescence, consistent with their weak absorbance profiles. A similar pattern was observed when the chromophores were excited at their maximum absorption wavelengths, where only 3 out of 6 chromophores showed fluorescence emission capabilities, as shown in Fig. S1b (ESI†).

**Fig. 2:**
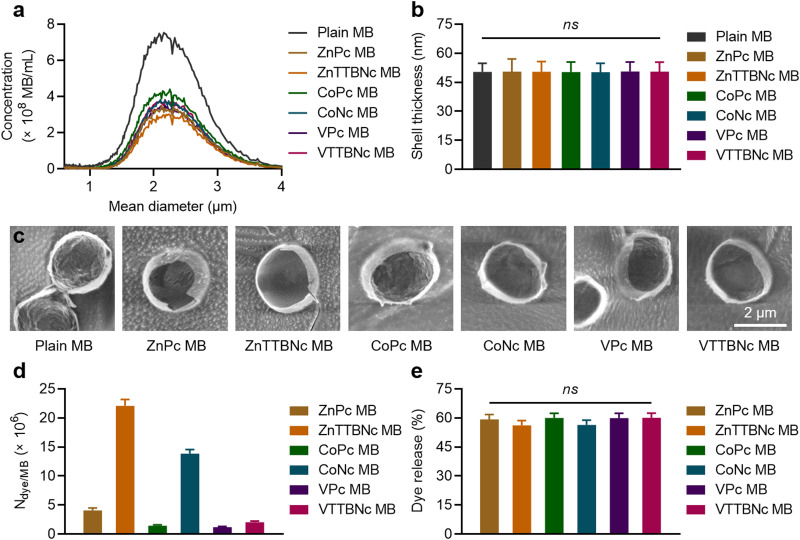
Metal phthalocyanine and naphthalocyanine encapsulation into PBCA MB. (a) Concentrations and diameter distributions of intact MB (Plain MB) and MB encapsulated with different chromophores (ZnPc MB, ZnTTBNc MB, CoPc MB, CoNc MB, VPc MB, and VTTBNc MB). (b) Shell thickness measurements of intact and chromophore-encapsulated MB by cryoSEM images. (c) Representative cryoSEM images of MB samples. (d) Chromophore encapsulation into MB shells as number of dye molecules loaded per one MB (*N*_dye/MB_). (e) US-induced chromophore release (%) from MB templates. ns indicates groups that are not significantly different with *p* > 0.05 (one-way ANOVA with *post hoc* Tukey HSD test). Representative wide-area OM and cryoSEM images, absorption spectra of MB samples, and chromophore loading per 1 × 10^9^ MB are presented in Fig. S2–S4 and Table S2 (ESI†), respectively.

Once the optical characteristics of the different chromophores were characterized, we tested their loading into PBCA MB shells. Based on our previous experience with hydrophobic molecules, we hypothesized that those chromophores with higher log *P* values would display higher loading efficiencies. The different MePc and MeNc chromophores were encapsulated in PBCA MB shells using a post-loading procedure. Fig. 2(a) shows the MB diameter distribution profiles of chromophore-encapsulated MB and plain PBCA MB (labeled as Plain MB), and demonstrates that the MB mean diameter was not affected by the loading procedure. The MB mean diameter was around 2.3 μm, which was consistent with our previous studies.^25,35^ The narrow diameter distributions of the MB samples were additionally confirmed by wide-area OM (Fig. S2, ESI†). Fig. 2(a) also shows a decrease in MB concentration after encapsulation, which can be attributed to the multiple washing steps associated with the post-loading procedure. Moreover, all MB samples showed similar shell thickness values, around 50 nm (Fig. 2(b)). Corresponding cryoSEM images of plain MB and dye-encapsulated MB further corroborated that the loading process did not affect the MB diameter and morphology, as observed in Fig. 2(c) and Fig. S3 (ESI†). Absorption (extinction) spectra of MB samples showed high turbidity (*i.e.*, strong light scattering) by the micron-sized templates (Fig. S4, ESI†), which was consistent with previous reports.^46,47^ Taken together, these results indicate that MePc and MeNc encapsulation does not affect the MB diameter distribution and shell thickness parameters. This is important, since variations in MB size and shell characteristics may alter (or even hamper) the MB acoustic properties.^35,48^

The efficiency of dye encapsulation dictates the PA contrast capabilities of the resulting platform, as higher levels of chromophores encapsulated in the MB shell are likely to provide stronger optical properties. Fig. 2(d) shows the values of dye molecules encapsulated per single MB (*N*_dye/MB_) for each sample, and Table S2 (ESI†) provides chromophore loading amounts per 1 × 10^9^ MB. The encapsulation values were proportional to the log *P* values of the molecules, with more hydrophobic MeNc chromophores displaying higher loading. When comparing encapsulation levels of MeNc or MePc, zinc(ii) chelated dyes were more efficiently encapsulated than cobalt(ii) chelated dyes, while vanadyl(iv) containing dyes showed the lowest encapsulation. Although these observations are likely caused by multiple factors, we hypothesize that the stable dimerization of vanadyl(iv) chelated chromophores with the formation of strong interplanar vanadyl–vanadyl or vanadyl–nitrogen bonds may have contributed to its lower loading in PBCA MB shells, as larger dye dimers may be harder to encapsulate than monomer chromophores. This hypothesis is consistent with the lower dimerization constants of ZnPc and CoPc compared to those of VPc.^49,50^ Overall, ZnTTBNc and CoNc showed the highest encapsulation rates with values of 2.2 ± 0.1 × 10^7^ and 1.4 ± 0.1 × 10^7^ dye molecules per MB, respectively, which corresponds to values of 22.0 ± 1.1 and 13.3 ± 0.7 μg of dye molecules/1 × 10^9^ MB, respectively. Notably, although the encapsulation levels were rather different between samples, all MB showed a similar percentage of chromophore release upon US exposure, as exemplified in Fig. 2(e).

### 3.2. US and PA performance of MePc and MeNc encapsulated MB in phantoms

**Fig. 3:**
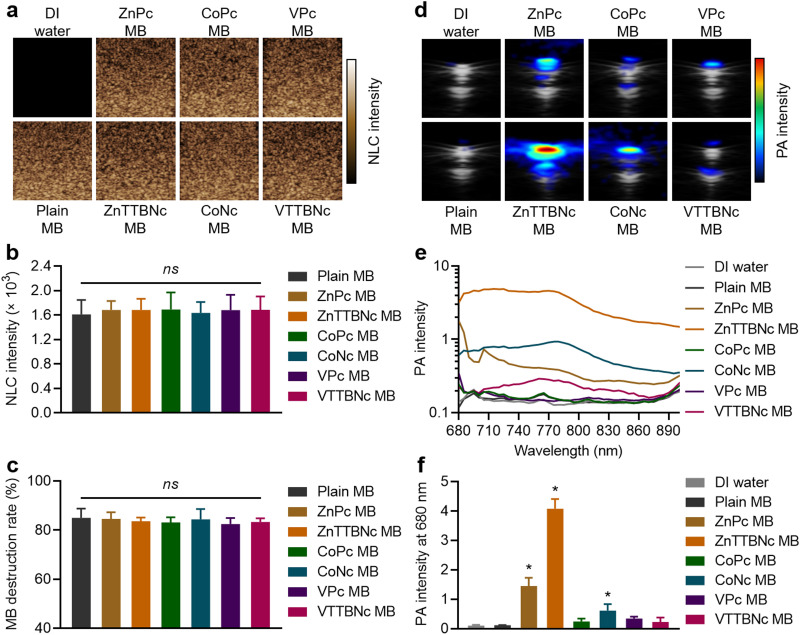
US and PA performance of chromophore-encapsulated MB in phantoms. (a) Representative US non-linear contrast (NLC) mode images of MB at 4% acoustic power. (b) Mean signal intensities of MB in NLC mode at 4% acoustic power. (c) MB destruction rates (%) of MB at 10% acoustic power. (d) Representative combined US B-mode and PA images of MB at 680 nm. (e) PA intensity spectra of MB recorded under excitation ranging from 680 to 900 nm. (f) Mean PA signal intensity of MB at 680 nm. Values represent mean ± standard deviation of three different batches of chromophore-encapsulated MB, measured in triplicates. All samples were measured at the same MB concentration (1.1 × 10^5^ MB mL^−1^ for US NLC, 1 × 10^9^ MB mL^−1^ for PA). ns and (*) indicate groups that are not significantly different with *p* > 0.05 and significantly different with *p* < 0.05 compared to the control groups (DI water and/or plain MB) (one-way ANOVA with *post hoc* Tukey HSD test), respectively.

While US imaging requires the acoustic responsiveness of the MB, PA imaging requires the MB to retain the PA characteristics of the encapsulated chromophores. Therefore, we evaluated the acoustic and PA response of chromophore-encapsulated MB formulations. Fig. 3(a) shows the sonographic images of the different samples in US non-linear contrast (NLC) mode, which is specific to MB acoustic responses under US pulses. While DI water (used as a control) did not provide any NLC signal, MB samples showed significant signal, which was characteristic of gas-filled MB. All (plain and chromophore-encapsulated) MB samples exhibited the same mean NLC intensity at 4% acoustic power (Fig. 3(b)) and similar degrees of MB destruction, around 80%, at 10% US acoustic power (Fig. 3(c)), consistent with our previous studies with PBCA MB of similar composition and diameter distribution.^25^

Fig. 3(d) shows combined US bright (B)-mode and PA images of MePc and MeNc encapsulated MB (and DI water and Plain MB taken as controls) obtained with the Vevo LAZR system at a wavelength of 680 nm and MB concentration of 1 × 10^9^ MB mL^−1^ in a vessel-like phantoms. While DI water and plain MB did not provide PA signal, all chromophore-encapsulated MB displayed PA signal, which varied among the different chromophore formulations. The PA spectra were recorded from 680 to 900 nm to characterize the PA response of the samples (Fig. 3(e)). Among the MB samples, ZnTTBNc MB displayed the highest PA intensity within the recorded wavelength range, with a PA signal intensity of 4.1 ± 0.3 at 680 nm (Fig. 3(f)), which was caused by the chromophore strong absorption band in the near-infrared region of the spectrum (centered at 764 nm) and its high hydrophobicity (log *P* value of 10.2) that resulted in high encapsulation values. Interestingly, CoNc MB displayed moderate PA signal, with an intensity of 0.6 ± 0.2 at 680 nm (Fig. 3(f)). Its highest PA signal was 0.9 ± 0.2 at 770 nm, despite having relatively low (compared to other chromophores) absorption in the red and near-infrared region of the spectrum. Its PA response was driven by the high hydrophobicity of the chromophore (log *P* value of 8.7), which yielded a high encapsulation efficiency. ZnPc MB also showed moderate PA response with a signal intensity of 1.4 ± 0.3 at 680 nm, which was likely caused by the balance between the strong absorbance of the chromophore and its relatively low hydrophobicity (log *P* value of 4.4) compared to MeNc, which resulted in moderate encapsulation levels. Despite the relatively poor encapsulation level of VTTBNc compared to other MeNc, its strong absorption near 800 nm promoted the weak but measurable PA signal intensity of VTTBNc MB over the spectral range. Considering the US and PA capabilities demonstrated by ZnTTBNc MB, CoNc MB and ZnPc MB, these three formulations were selected for further characterization.

**Fig. 4:**
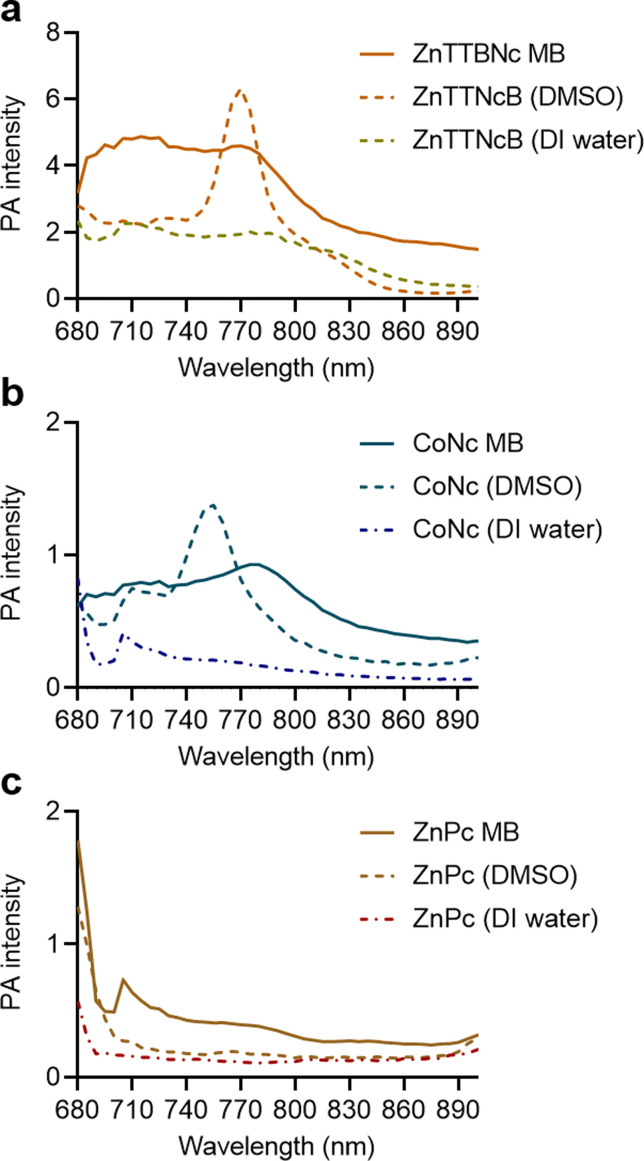
Comparison of PA performance of chromophore-encapsulated MB *versus* chromophores dissolved in DMSO and DI water at equal concentrations in phantoms. (a) ZnTTBNc-, (b) CoNc-, and (c) ZnPc-containing samples were adjusted to the concentrations presented in Table S2 (ESI†), and PA intensities were recorded in a wavelength range from 680 to 900 nm.

Next, we compared the PA performances of the selected MB formulations and the free chromophores dissolved in either DMSO or DI water. ZnTTBNc MB, CoNc MB and ZnPc MB samples showed higher PA signal intensities than the free chromophore counterparts in water, as shown in Fig. 4. We hypothesize that the MB formulations performed better because of two factors. First, the MePc and MeNc loaded inside the shell were protected from water-induced aggregation and PA signal quenching. Second, the oscillations produced by the MB shell might have synergized with the thermoelastic expansion of the chromophores, improving the PA performances of the chromophore-loaded MB samples. This synergistic effect has been recently reported in other formulations, such as MB loaded with porphyrin dyes and gold nanoparticles.^30–32^ While chromophores dissolved in DMSO could yield higher PA intensities around their maximum absorption bands, dye-loaded MB could exceed the PA performances of the chromophores over a wider range of wavelengths. These bandwidth increases may be related to the local aggregation of the dyes in the shells during the loading procedure (similar to a previous report^18^). However, these potential local aggregations did not quench the PA responses and provided wider PA responses over multiple wavelengths, which combined with the water solubility of the MB formulations yielded highly performing PA probes. In addition, all selected MB samples preserved PA stability over longer exposure times (3 min) as shown in Fig. S6 (ESI†).

Furthermore, although the PA bands of the selected MB samples were broad, they showed a significant PA contrast signal at multiple frequencies in the red and near-infrared spectral regions, which can be distinguished from numerous endogenous and clinically relevant PA chromophores, including oxy- and deoxy-hemoglobin, and collagen.^1,6,51^ Therefore, the described probes could be potentially used for multispectral PA imaging, where the tissue of interest is illuminated with a set of excitation wavelengths relevant to each specific (endogenous or exogenous) chromophore.

Next, the colloidal and PA signal stability of selected MB samples was evaluated in biological fluids, *i.e.*, in solutions containing 10% and 50% FBS (Fig. S7 and S8, ESI† respectively). When ZnTTBNc MB were transferred to PBS and mixed with 10% FBS, the diameter distribution of the samples remained unchanged (Fig. S7a, ESI†), preserving the mean diameter around 2.3 μm over 24 h (Fig. S7b, ESI†). The stability of the MB also yielded strong PA signals up to 24 h, as shown in the PA intensity spectra (Fig. S7c, ESI†) and the mean PA signal intensity at 770 nm (Fig. S7d, ESI†). Similar patterns of preserved colloidal and PA signal stability were observed for CoNc MB and ZnPc MB as shown in Fig. S7e–h and i–l (ESI†), respectively. When the FBS concentration was increased to 50%, the mean diameter of the MB samples remained intact for 30 min, while the diameter slightly decreased at the 2 h time point and then remained stable up to 24 h, as shown in Fig. S8a, e, and i (ESI†) for ZnTTBNc MB, CoNc MB, and ZnPc MB, respectively. We hypothesize that this was caused by the potential stiffening of the MB shell during the protein corona formation, leading to partial dissolution of the MB gaseous core to equilibrate the osmotic pressure with the Laplace pressure of the MB. This is in agreement with previous reports on MB dissolution models.^52–54^ However, such decreases in mean diameter were not statistically significant, as demonstrated in Fig. S8b, f, and j (ESI†), for ZnTTBNc MB, CoNc MB, and ZnPc MB, respectively. Hence, the stability and strong PA signal of chromophore-encapsulated MB were preserved over 24 h.

**Fig. 5:**
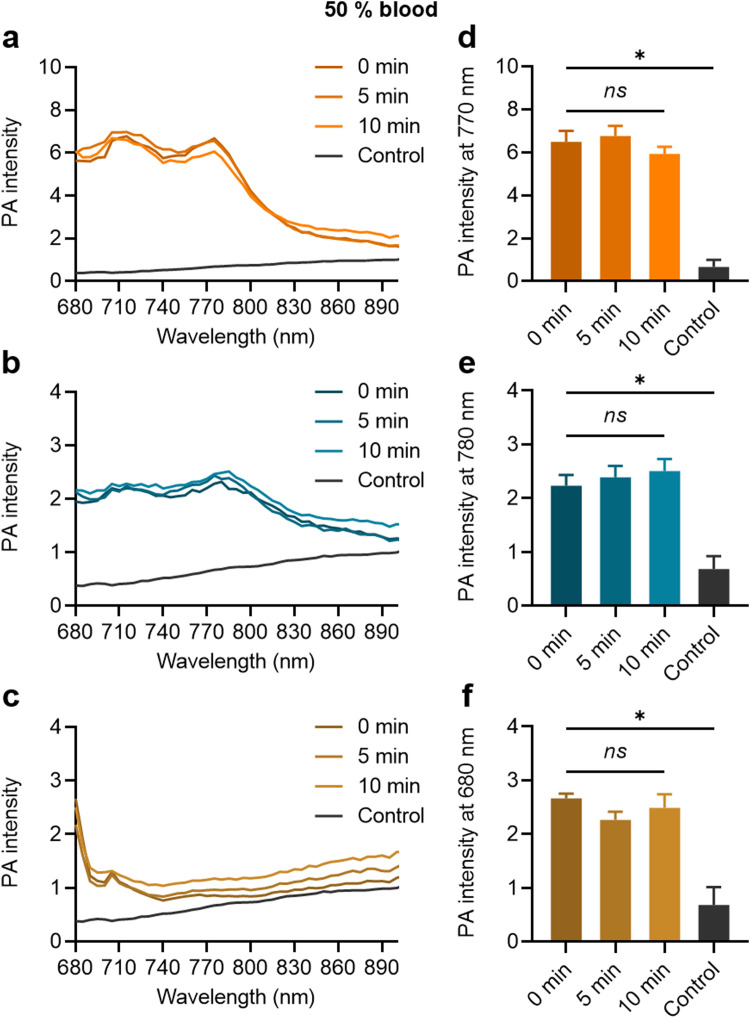
PA signal stability in 50% blood solution. (a)–(c) PA intensity spectra recorded under excitation ranging from 680 to 900 nm, and (d)–(f) mean PA signal intensities at specified wavelegnths of ZnTTBNc MB, CoNc MB, and ZnPc MB, respectively. Samples were evaluated at specified time points within 10 min. ns and (*) indicate groups that are not significantly different with *p* > 0.05 and significantly different with *p* < 0.05 compared to the control group (one-way ANOVA with *post hoc* Tukey HSD test), respectively.

When the MB samples were dispersed in the 50% blood solution, their strong PA contrast signals were clearly distinguishable from the control, as shown in Fig. 5(a)–(c) for ZnTTBNc MB, CoNc MB, and ZnPc MB, respectively. The MB contrast signal remained stable over the tested time (up to 10 min), as shown by the PA signal intensities at the characteristic PA bands of ZnTTBNc, CoNc, and ZnPc (*i.e.*, 770, 780, and 680 nm, respectively) compared to the control (Fig. 5(d)–(f), respectively). The testing time in blood was selected based on the *in vivo* circulation time of PBCA MB after systemic administration, which is inferior to 10 min.^36^ Taken together, the MB samples retained colloidal stability and strong PA signals that were clearly distinguishable from the background of the biofluids.

### 3.3. *Ex vivo* proof-of-concept US/PA imaging

**Fig. 6:**
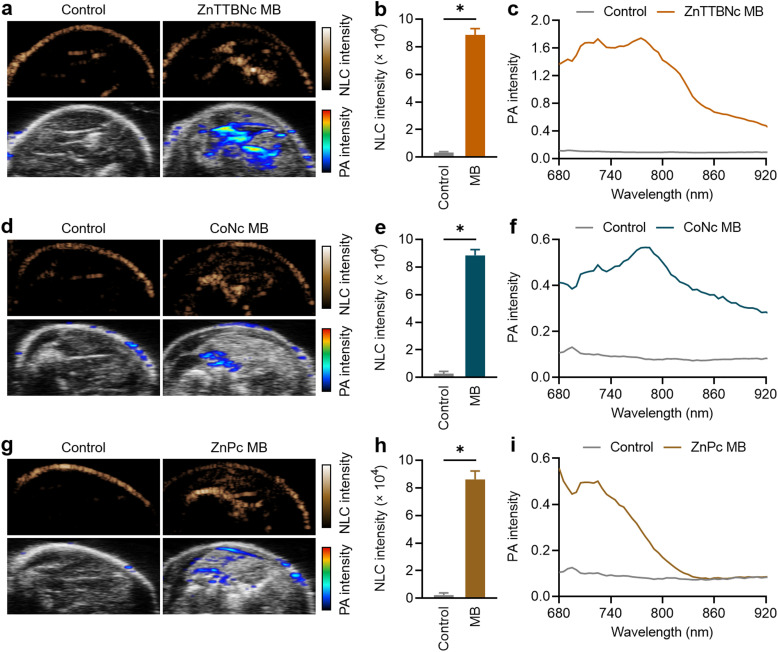
*Ex vivo* US/PA imaging. (a) Representative images in US NLC mode, and overlays of US B-mode with PA at 680 nm, (b) mean US NLC intensities, and (c) mean PA spectra before and after intramuscular injection of ZnTTBNc MB (referred to as control and ZnTTBNc MB, respectively). (d) Representative US and PA images, (e) mean NLC intensities, and (f) mean PA spectra before and after intramuscular injection of CoNc MB. (g) Representative US and PA images, (h) mean NLC intensities, and (i) mean PA spectra before and after intramuscular injection of ZnPc MB. (*) indicates groups that are significantly different with *p* < 0.05 (one-way ANOVA with *post hoc* Tukey HSD test).

For *ex vivo* proof-of-concept US/PA imaging, legs of deceased mice were intramuscularly injected with 1 × 10^9^ MB of each selected sample. This PBCA MB dose has been widely used in numerous *in vivo* studies for US imaging and therapy, and it has been demonstrated to be safe.^28,36,55,56^ Regarding the MePc and MeNc, previous studies proved that the chromophores did not induce any dark cytotoxicity at concentrations as high as 400 μg mL^−1^,^57–61^ which were 18 to 100 times higher than the amounts of chromophores encapsulated in the MB samples and injected during the *ex vivo* experiments (as indicated in Table S2, ESI†). Fig. 6(a) shows representative images of the mouse leg before and after ZnTTBNc MB injection (control and ZnTTBNc MB images, respectively) in US NLC and PA modes. The PA signals are overlaid with the US B-mode sonographic images for clarity. The administration of ZnTTBNc MB resulted in strong signal intensities in both the US NLC mode (related to the acoustic features of the MB formulation) and PA mode (based on the PA capabilities of the ZnTTBNc encapsulated MB). A small signal on the skin surface could be observed in both imaging modalities, likely related to the sound scattering at the tissue boundary in the US NLC mode and the presence of melanin (and other endogenous chromophores) in the skin in the PA mode.^62^ In addition, a small acoustic shadowing under the areas filled with MB may be observed on the US B-mode images due to a high local concentration of MB.^63^Fig. 6(b) provides a quantitative assessment of the mean US NLC intensities before and after ZnTTBNc MB injection, highlighting the bright signal provided by the formulation (up to 8850 ± 440 c.u.) compared to the signal recorded before injection in the same region of interest (around 280 ± 120 c.u.). PA spectra were recorded before and after ZnTTBNc MB injection in the wavelength range from 680 to 920 nm (Fig. 6(c)), showing significant mean PA intensities provided by the formulation. Values up to 1.75 were obtained at 770 nm, which correlated well with the absorbance band of ZnTTBNc. Similarly, strong signal enhancements were observed in both US NLC and PA modes after CoNc MB and ZnPc MB injection (Fig. 5(d) and (g), respectively). The NLC intensities of both samples were similar to the one recorded for ZnTTBNc MB with values of 8830 ± 420 c.u. for CoNc MB (Fig. 6(e)) and 8610 ± 600 c.u. for ZnPc MB (Fig. 6(h)), since all MB displayed similar sizes and shell thickness. The PA spectra of the tissues injected with CoNc MB and ZnPc MB showed moderate increases in signal (up to 0.57 ± 0.11 at 770 nm for CoNc MB and 0.56 ± 0.06 at 680 nm for ZnPc MB) compared to the same tissues before contrast administration (0.10 ± 0.05 at 680 and 770 nm) (Fig. 6(f) and (i), respectively). Taken together, the US and PA responses *ex vivo* of the selected formulations were consistent with the ones obtained *in vitro*. While the three chromophore-loaded MB displayed similar US performances, ZnTTBNc MB was the formulation that displayed the strongest PA signal.

Lastly, since the NLC and PA intensities were very intense for the ZnTTBNc MB, we measured whether their concentration could be reduced by a factor of 4 (*i.e.*, to 2.5 × 10^8^ MB) and still display strong responses in both US and PA imaging modalities. At those low concentrations, ZnTTBNc displayed high intensities both in US NLC and PA images (Fig. S9a, ESI†), with NLC intensity as high as 1332 ± 34 c.u. after MB injection compared to 110 ± 8 c.u. of the control (Fig. S9b, ESI†), and PA intensities of 0.65 and 0.87 at 680 nm and 770 nm (Fig. S9c, ESI†), respectively, which exceeded the intensities of CoNc MB and ZnPc MB samples at four times higher concentrations.

Taken together, the results presented in this study demonstrate that the capabilities of PBCA MB can be further expanded by loading their shell with metal phthalocyanines. The resulting formulation show both US and PA imaging characteristics, which may provide new avenues to acquire diagnostic and therapeutic information. We envision the multifunctional contrast agents being used in different ways, including discerning the response of intact PBCA MB (US) and the release of cargo (PA) upon irradiation with US destructive pulses. Moreover, if metal phthalocyanines were combined with other chromophores with different spectral characteristics, multiplexing imaging could be developed, where several MB formulations loaded with different chromophores and functionalized with distinct targeting agents could be used to simultaneously differentiate multiple vascular biomarkers.

## 4. Conclusions

In summary, we demonstrate that PBCA MB encapsulated with metal phthalocyanines constitute a versatile and robust platform for multimodal US and PA imaging. The higher hydrophobicity of the metal naphthalocyanines promotes higher chromophore loading into the MB polymer shells compared to their phthalocyanine counterparts. Notably, after loading, the resulting formulations maintain the chromophore optical properties required for PA imaging. Among the different metal chromophores, zinc and cobalt chelated naphthalocyanines show the highest degree of encapsulation into MB shells, with values up to 2.2 × 10^7^ dye molecules per MB, yielding formulations with intense US NLC and PA responses *in vitro*. The strong imaging capabilities of the MB formulations are preserved in biological environment, as demonstrated *in vitro* in serum and whole blood, and *ex vivo* in deceased mice. These findings highlight the advantages of the rational design of hybrid contrast agents based on US-responsive polymeric MB and highly hydrophobic PA contrast agents, which may be beneficial for multifunctional imaging and monitoring of drug release.

## Abbreviations

PA: Photoacoustic
US: Ultrasound
MB: Microbubbles
PBCA: Poly(butyl cyanoacrylate)
MePc: Metal phthalocyanines
MeNc: Metal naphthalocyanines
ZnPc: Zinc(ii) phthalocyanine
ZnTTBNc: Zinc(ii) 2,11,20,29-tetra-*tert*-butyl-2,3-naphthalocyanine
CoPc: Cobalt(ii) phthalocyanine (CoPc)
CoNc: Cobalt(ii) 2,3-naphthalocyanine
VPc: Vanadyl(iv) phthalocyanine (VPc)
VTTBNc: Vanadyl(iv) tetra-*tert*-butyl-2,3-naphthalocyanine (VTTBNc)
NLC: Non-linear contrast.

## Conflicts of interest

F. Kiessling, and T. Lammers are among the co-founders of the SonoMAC GmbH that produces polymeric microbubbles. F. Kiessling is a consultant of Fujifilm Visualsonics.

## Supplementary Material

TB-012-D3TB02950F-s001
