# Supplementary material for: Hybrid ultrasound and photoacoustic contrast agent designs combining metal phthalocyanines and PBCA microbubbles
Source: J Mater Chem B. 2024 Feb 9;12(10):2511–22. doi: 10.1039/d3tb02950f (PMC10916536; doi:10.1039/d3tb02950f)
Supplement: TB-012-D3TB02950F-s001 [file TB-012-D3TB02950F-s001.pdf]

Electronic Supplementary Material (ESI) for Journal of Materials Chemistry B.
This journal is © The Royal Society of Chemistry 2024

**- SUPPORTING INFORMATION -**

# Hybrid Ultrasound and Photoacoustic Contrast Agent Designs Combining Metal Phthalocyanines and PBCA Microbubbles

Roman A. Barmin,[1] MirJavad Moosavifar,[1] Rui Zhang,[1] Stephan Rütten,[2] Sven Thoröe-Boveleth,[3] Elena Rama,[1] Tarun Ojha,[1] Fabian Kiessling,[1] Twan Lammers,[1,*] and Roger M. Pallares[1,*]

[1] Institute for Experimental Molecular Imaging, RWTH Aachen University Hospital, Aachen 52074, Germany

[2] Electron Microscope Facility, RWTH Aachen University Hospital, Aachen 52074, Germany

[3] Institute for Occupational, Social and Environmental Medicine, RWTH Aachen University Hospital, Aachen 52074, Germany

* Corresponding authors: rmoltopallar@ukaachen.de; tlammers@ukaachen.de

# Table of Contents

Table S1. Literature values of maximum absorption wavelengths, molar absorption coefficients, and fluorescence quantum yields of selected chromophores ....................... S3

Figure S1. Optical properties of metal phthalocyanines and naphthalocyanines in DI water and DMSO ........................................................................................ S4

Figure S2. Representative wide-area OM micrographs of MB samples .......................... S5

Figure S3. Representative wide-area cryoSEM micrographs of MB samples ................. S6

Figure S4. Absorption (extinction) spectra of MB samples ............................................ S7

Table S2. Chromophore loading per $1 \times 10^9$ MB ........................................................... S8

Figure S5. PA intensity spectra of the different chromophores dissolved in DMSO at the same amounts as loaded per $1 \times 10^9$ MB ............................................................... S9

Figure S6. PA signal stability of MB over time recorded at 680 nm ............................ S10

Figure S7. Colloidal and PA signal stability of chromophore-encapsulated MB in 10 % FBS solution ........................................................................................................ S11

Figure S8. Colloidal and PA signal stability of chromophore-encapsulated MB in 50 % FBS solution ........................................................................................................ S12

Figure S9. *Ex vivo* US/PA imaging at low ZnTTBNc MB concentration...................... S13

References..................................................................................................................... S14

**Table S1.** Literature values of maximum absorption wavelengths ($\lambda_{abs}$), molar absorption coefficients ($\mathcal{E}$), and fluorescence quantum yields ($\Phi_F$) of selected chromophores.

| **Chromophore** | **$\lambda_{abs}$ (nm)** | **$\varepsilon$ (× $10^5$ L / $M^{-1}$ $cm^{-1}$)** | **$\Phi_F$** | **Solvent** | **Ref.** |
|---|---|---|---|---|---|
| ZnPc | 672 | 2.40 | 20 % | DMSO | 1,2 |
| ZnTTBNc | 766 | 1.74 | 7 % | DMSO | 1,3 |
| CoPc | 660 | 1.10 | – | Pyridine | 2,4 |
| CoNc | 752 | 1.53 | – | Pyridine | 4 |
| VPc | 698 | 1.02 | – | Pyridine | 4 |
| VTTBNc | 810* | 1.26* | < 2 %** | Pyridine,* toluene** | 3–5 |

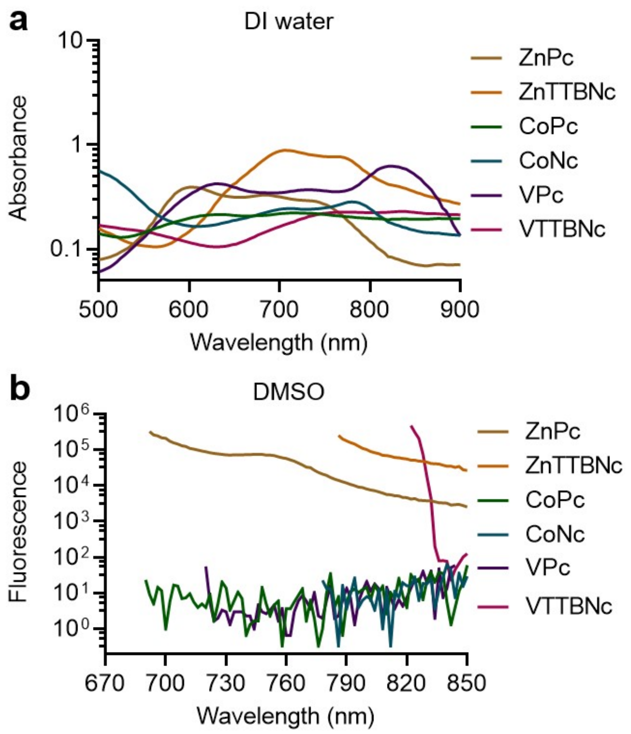


**Figure S1. Optical properties of metal phthalocyanines and naphthalocyanines in DI water and DMSO.** (**a**) Absorption spectra of the different chromophores in DI water, and (**b**) fluorescence emission spectra of the different chromophores in DMSO upon excitation at the maximum absorption wavelengths shown in Table S1.

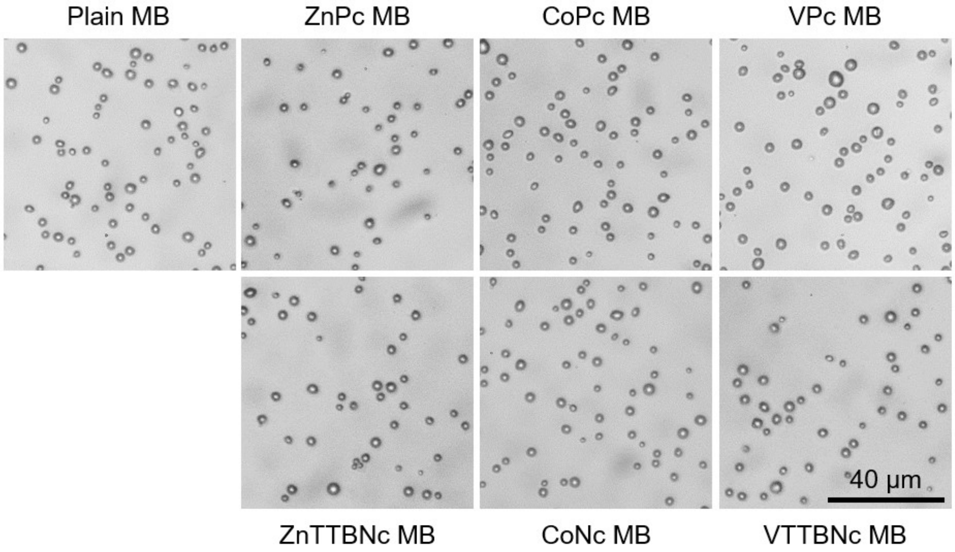


**Figure S2. Representative wide-area OM micrographs of MB samples.**

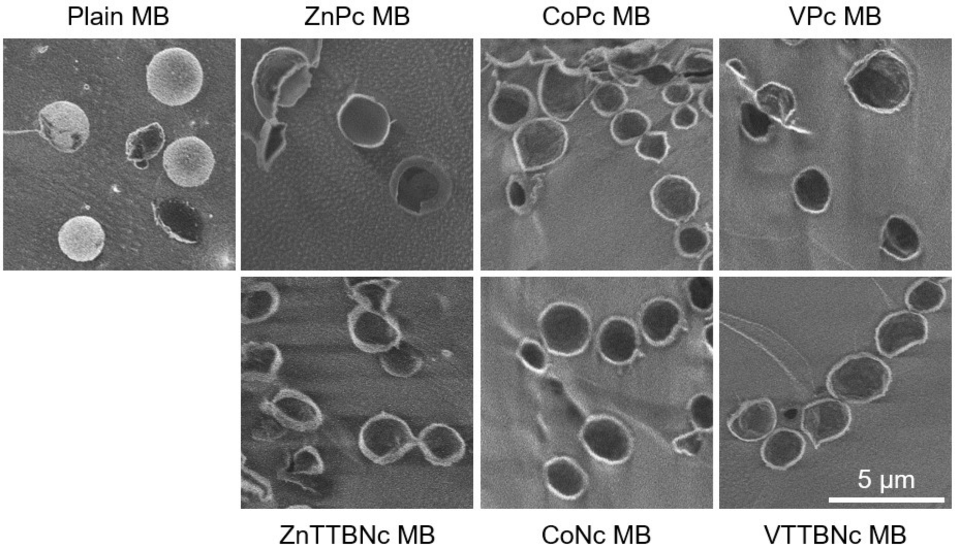


**Figure S3. Representative wide-area cryoSEM micrographs of MB samples.**

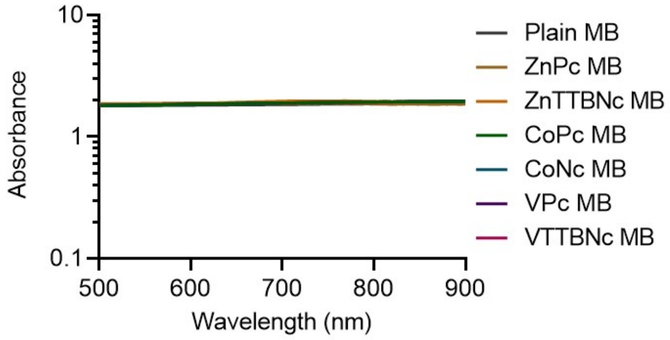


**Figure S4. Absorption (extinction) spectra of MB samples.**

**Table S2. Chromophore loading per 1 × $10^9$ MB.**

| Sample | Chromophore loading (µg / 1 × $10^9$ MB) |
|---|---|
| ZnPc MB | 3.9 ± 0.4 |
| ZnTTBNc MB | 22.0 ± 1.1 |
| CoPc MB | 1.4 ± 0.2 |
| CoNc MB | 13.3 ± 0.7 |
| VPc MB | 1.2 ± 0.1 |
| VTTBNc MB | 2.0 ± 0.2 |

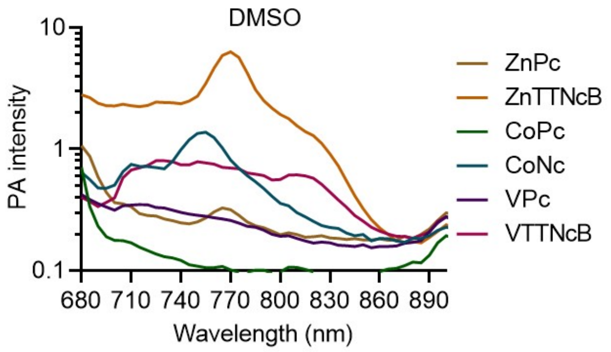


**Figure S5. PA intensity spectra of the different chromophores dissolved in DMSO at the same amounts as loaded per 1 × $10^9$ MB.** Spectra of all samples were recorded in the wavelength range of 680 to 900 nm.

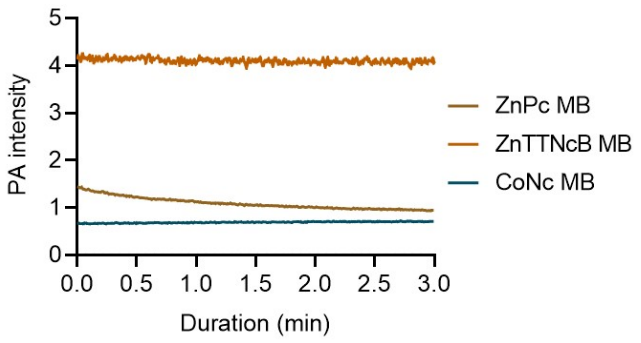


**Figure S6. PA signal stability of MB over time recorded at 680 nm.**

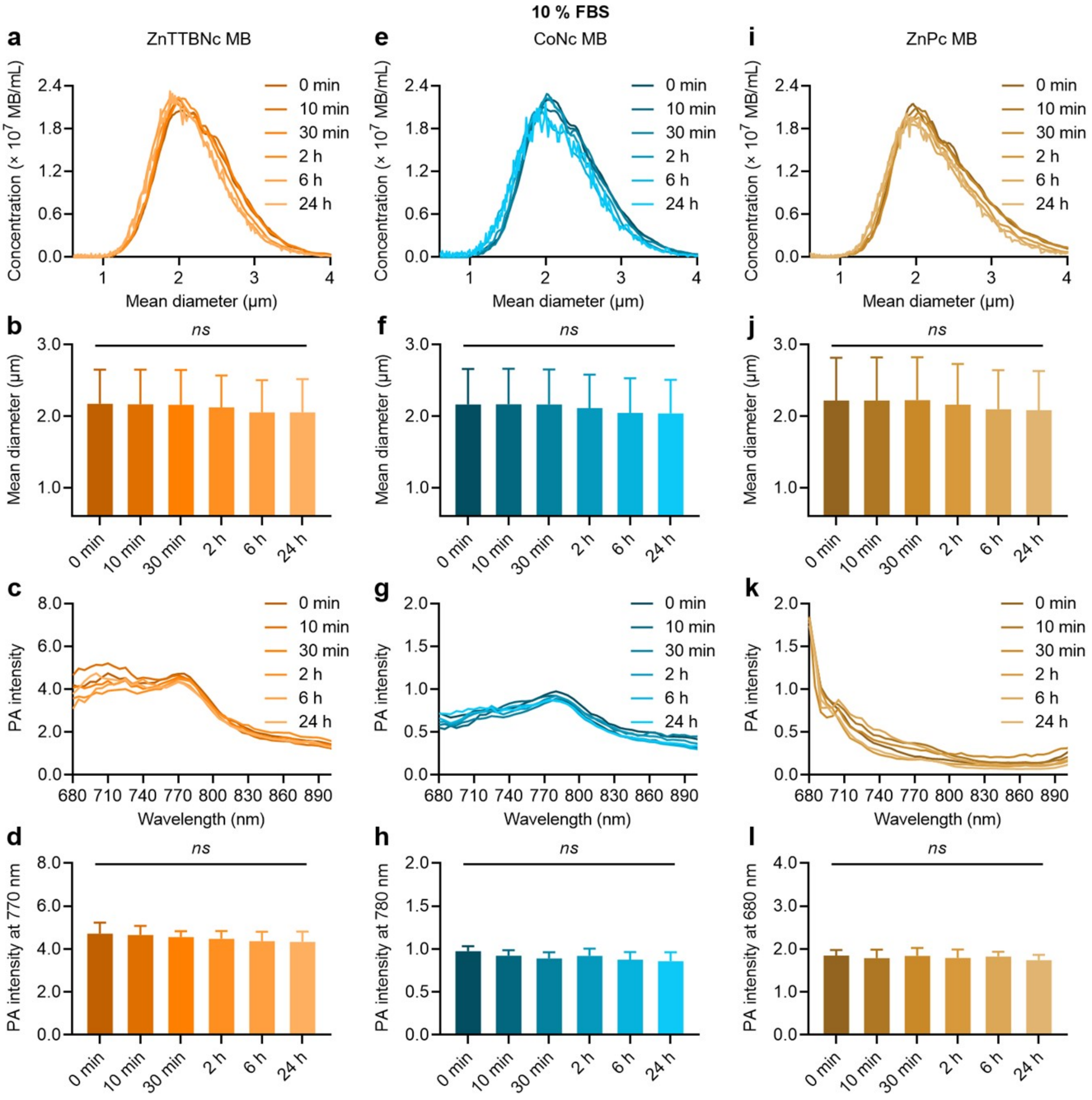


**Figure S7. Colloidal and PA signal stability of chromophore-encapsulated MB in 10 % FBS solution.** (**a, e, i**) Concentrations and diameter distributions, (**b, f, j**) mean diameters, (**c, g, k**) PA intensity spectra recorded under excitation ranging from 680 to 900 nm, and (**d, h, l**) mean PA signal intensities at specified wavelegnths of ZnTTBNc MB, CoNc MB, and ZnPc MB, respectively. Samples were evaluated at specified time points within 24 h. *ns* indicates groups that are not significantly different with $p > 0.05$ (one-way ANOVA with post hoc Tukey HSD test).

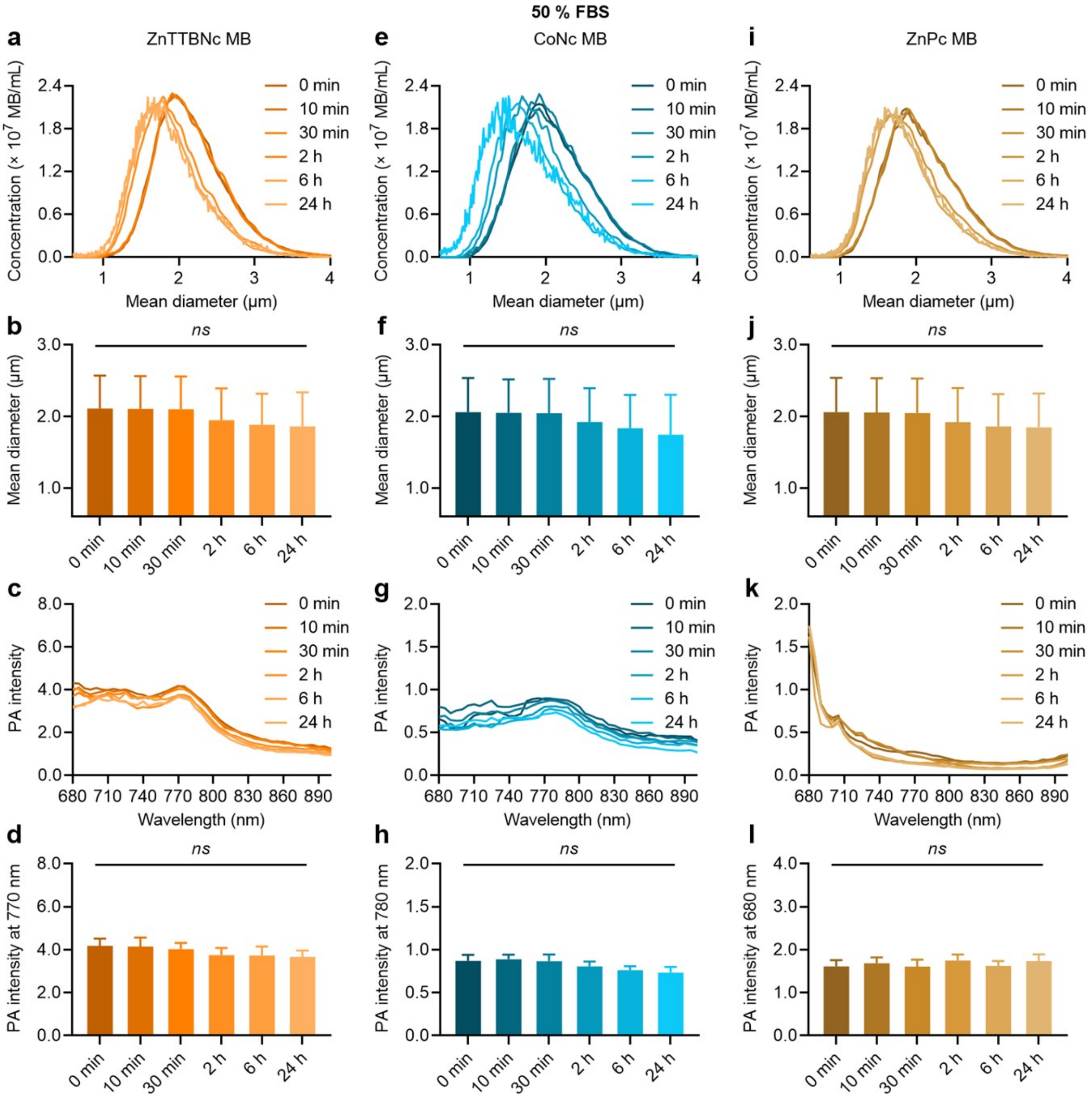


**Figure S8. Colloidal and PA signal stability of chromophore-encapsulated MB in 50 % FBS solution.** (**a, e, i**) Concentrations and diameter distributions, (**b, f, j**) mean diameters, (**c, g, k**) PA intensity spectra recorded under excitation ranging from 680 to 900 nm, and (**d, h, l**) mean PA signal intensities at specified wavelegnths of ZnTTBNc MB, CoNc MB, and ZnPc MB, respectively. Samples were evaluated at specified time points within 24 h. *ns* indicates groups that are not significantly different with $p > 0.05$ (one-way ANOVA with post hoc Tukey HSD test).

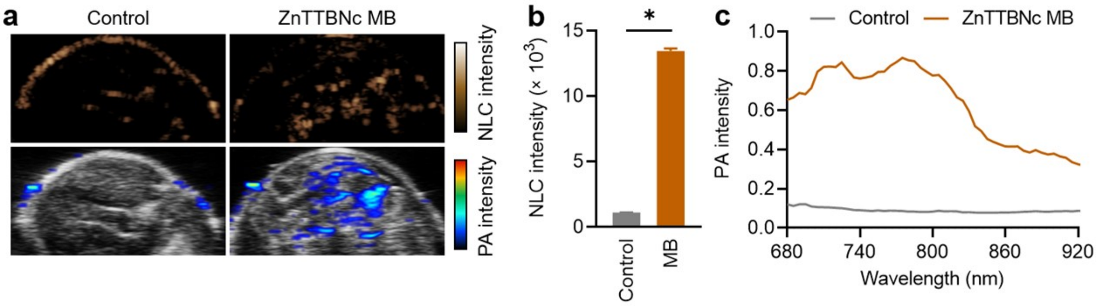


**Figure S9. *Ex vivo* US/PA imaging at low ZnTTBNc MB concentration.** (**a**) Representative images in US NLC, and overlays of US B-mode with PA at 680 nm, (**b**) mean US NLC intensities, and (**c**) mean PA spectra before and after intramuscular injection of ZnTTBNc MB (labeled as control and ZnTTBNc MB, respectively). (*) indicates groups that are significantly different with $p < 0.05$ (one-way ANOVA with post hoc Tukey HSD test).

**REFERENCES**


1 A. Ogunsipe, J.-Y. Chen and T. Nyokong, *New J. Chem.*, 2004, **28**, 822–827.

2 F. Ghani, J. Kristen and H. Riegler, *J. Chem. Eng. Data*, 2012, **57**, 439–449.

3 Y. Zhang, M. Jeon, L. J. Rich, H. Hong, J. Geng, Y. Zhang, S. Shi, T. E. Barnhart, P. Alexandridis, J. D. Huizinga, M. Seshadri, W. Cai, C. Kim and J. F. Lovell, *Nat. Nanotechnol.*, 2014, **9**, 631–638.

4 N. Kobayashi, S. Nakajima, H. Ogata and T. Fukuda, *Chem. – A Eur. J.*, 2004, **10**, 6294–6312.

5 M. J. Duffy, O. Planas, A. Faust, T. Vogl, S. Hermann, M. Schäfers, S. Nonell and C. A. Strassert, *Photoacoustics*, 2018, **9**, 49–61.
